# Comparison of Pain and Complications between Outpatients and Inpatients Treated with Bone Marrow Aspirate Concentrate for Knee Osteoarthritis

**DOI:** 10.3390/jpm14090942

**Published:** 2024-09-05

**Authors:** Ji-Hoon Baek, Su Chan Lee, Dong Nyoung Lee, Juneyoung Heo, Taehyeon Kim, Hye Sun Ahn, Chang Hyun Nam

**Affiliations:** 1 Joint & Arthritis Research, Department of Orthopaedic Surgery, Himchan Hospital, Seoul 07999, Republic of Korea; jihoon011@naver.com (J.-H.B.); himchanhospital@naver.com (S.C.L.); fretless@hanmail.net (D.N.L.); ysunispica@naver.com (T.K.); ahs0614@naver.com (H.S.A.); 2 Joint & Arthritis Research, Department of Neurosurgery, Himchan Hospital, Seoul 07999, Republic of Korea; juneyoungheo@gmail.com

**Keywords:** pain, complications, bone marrow aspirate concentrate, outpatients, inpatients

## Abstract

Bone marrow aspirate concentrate (BMAC) has been increasingly used as an injectable treatment for knee osteoarthritis (OA). However, there remains a lack of studies on the pain and complications associated with BMAC treatment. This study compared the pain and complications of BMAC treatment between outpatients and inpatients with Kellgren–Lawrence grade II–III knee osteoarthritis (OA) during a follow-up period of ≥3 months. This study included 40 outpatients (40 knees) and 80 inpatients (80 knees) as controls who received BMAC articular injections for knee OA between December 2023 and March 2024. Outpatients were administered BMAC under local anesthesia alone, whereas inpatients were administered BMAC under local anesthesia and intravenous anesthesia. The outcomes were the visual analog scale (VAS) pain score during the BMAC procedure and the complications associated with harvest and injection sites. The mean VAS pain score in the outpatient group was significantly higher than that in the inpatient group during trocar insertion (5.2 vs. 1.3, *p* < 0.05) and bone marrow aspiration (6.2 vs. 1.4, *p* < 0.05), but it was similar between the two groups during BMAC injection (2.2 vs. 2.3, *p* = 0.858). Transient post-treatment complications were observed in 17.5% (7/40) of outpatients and 16.3% (13/80) of inpatients. No significant differences were observed in complications between the two groups, all of which were resolved within 2 months without any specific problem. Moreover, no major complications occurred in any group. In conclusion, outpatients who received only local anesthesia reported significant pain during BMAC treatment. The addition of intravenous anesthesia is necessary to alleviate pain during the BMAC procedure.

## 1. Introduction

Osteoarthritis (OA) is a chronic inflammatory and degenerative joint disease in adults that leads to irreversible deterioration and the loss of articular cartilage, resulting in a significant socioeconomic burden [1,2]. The knee is particularly affected, with the incidence of OA continuing to increase [3]. Nonsurgical treatments for knee OA include medications, physical therapy, weight loss, and intra-articular injections. If nonsurgical options fail and the condition progresses to end-stage knee OA, total knee arthroplasty can be an effective alternative treatment [4]. However, this procedure has some complications, as reported in a previous study, wherein loss of function or persistent pain was observed in 20% of patients even 1 year after surgery [5,6]. Consequently, biological therapies such as stem cell therapy, which has the potential to alter the natural course of OA treatment, have attracted increasing attention in recent years [7].

Bone marrow aspirate concentrate (BMAC) has been increasingly used as an injectable treatment for knee OA. In Korea, the use of BMAC increased after its approval by the Ministry of Health and Welfare in July 2023. Since 2013, the feasibility and safety of BMAC treatment for knee OA have been confirmed in several randomized controlled trials, demonstrating clinical efficacy and significant functional improvement [8,9,10]. BMAC treatment involves four main steps: harvest needle insertion, bone marrow aspiration, bone marrow centrifugation, and BMAC injection into the patient’s knee. To date, most studies on BMAC treatment for knee OA have focused on its clinical efficacy, with few of them addressing associated pain and complications at harvest and injection sites [11]. In addition, most of the studies typically used local anesthesia with lidocaine for BMAC treatment as an outpatient procedure. However, based on our experience in BMAC treatment over several months, patients experience significant pain during the procedure, accompanied by several complications. Therefore, with the increasing use of BMAC treatment in patients with OA, it is necessary to manage the pain and complications associated with the procedure.

To the best of our knowledge, there has been no discussion on the optimal setting (outpatient or inpatient) for BMAC treatment, nor on the optimal method for managing pain during bone marrow harvesting from the ilium (using local anesthesia alone or in combination with intravenous anesthesia). In other words, there is no universal protocol for the optimal therapeutic setting and prevention of pain during BMAC treatment. Therefore, this study compared and evaluated the pain and complications related to BMAC treatment between outpatients and inpatients with Kellgren–Lawrence (K–L) grade II–III knee OA. We hypothesized that outpatients treated with local anesthesia alone experience substantially more pain than inpatients treated with local anesthesia combined with intravenous anesthesia during BMAC treatment.

## 2. Materials and Methods

The Institutional Review Board of our hospital approved the design and protocol of this retrospective study and the review of medical records, as well as waiving the requirement for informed consent.

Furthermore, 605 patients (756 knees) with symptomatic degenerative knee OA received BMAC treatment at our institution between December 2023 and March 2024. The inclusion criterion for BMAC treatment was patients with K–L grade [12] II–III OA and chronic knee pain who had not responded to conservative treatment for at least 6 weeks. Exclusion criteria included patients with K–L grade I or IV degenerative arthritis based on the standards approved by the Ministry of Health and Welfare in Korea. Since the approval of BMAC treatment in July 2023, it has been performed under local anesthesia with lidocaine at our institution. However, after 3 months of experience, we noticed that many patients had concerns regarding the pain associated with BMAC treatment and experienced significant pain during the procedure. Therefore, we subsequently performed the procedure under intravenous anesthesia followed by local anesthesia at the harvest site during hospitalization. However, during the study period, 40 patients (40 knees; 8 men and 32 women) received BMAC treatment for one knee in an outpatient setting under local anesthesia alone because of personal circumstances (outpatient group). The outpatients were compared with the control group (80 inpatients) who received BMAC treatment under local and intravenous anesthesia during the study period. Propensity score matching was performed using a 1:2 ratio. Demographic data, including age, sex, body mass index (BMI), and K–L grade, were obtained from medical records. The mean follow-up periods in outpatient and inpatient groups were 4.6 ± 0.8 (range, 3–6) and 4.5 ± 1.0 (range, 3–6) months, respectively.

### 2.1. BMAC Harvest and Injection Procedure in the Inpatient Group

In the inpatient group, intravenous anesthesia with midazolam (0.035 mg/kg) was administered by an anesthesiologist to ensure sedation before performing the procedure. BMAC can be harvested from many sites; however, the anterior iliac crest ipsilateral to the knee for injection was selected in this study because it contains the highest number of osteogenic progenitor cells [13]. Therefore, all bone marrow harvests were performed on the anterior iliac crest.

The patient was placed in a supine position on the operating table. A bump was placed under the hip to expose the iliac crest, after which the surrounding area was disinfected with chlorhexidine and betadine and then draped. The anterior superior iliac spine (ASIS) was palpated; then, at 2–3 cm proximal to the ASIS, 2% lidocaine (5 mL) was injected into the area to be harvested using a syringe. After creating a 0.5 cm stab incision using a No. 11 scalpel at the harvest site, a BMAC harvest needle with a sharp trocar was inserted percutaneously. Using the trocar, the midpoint of the iliac crest was identified by palpating between the inner and outer cortices. Using a mallet, the BMAC harvest needle was advanced approximately 2 cm (two divisions on the scale) from the midpoint of the iliac crest between the inner and outer cortices and in a trajectory parallel to the iliac crest. The trocar was withdrawn, after which a heparin-coated syringe (containing 1 mL of heparin and 4 mL of normal saline) was used to collect bone marrow (60 mL). After suturing and disinfecting the harvest site, pressure was applied for 5 min to achieve hemostasis. The harvested bone marrow was transferred to a disposable sterile container and concentrated via single-spin centrifugation at 4000 rpm for 12 min to remove the plasma and erythrocyte components and obtain approximately 6 mL of BMAC, which was injected into the affected knee through the superolateral margin of the patella. After the intra-articular injection, the knee was flexed and extended several times to allow diffusion of the BMAC into the knee joint. The patient was hospitalized to observe the progress post-BMAC treatment and returned home the next day.

### 2.2. BMAC Harvest and Injection Procedure in the Outpatient Group

In the outpatient group, the procedure was conducted without intravenous anesthesia using midazolam. Bone marrow harvesting and BMAC injection were performed as described above, and only local anesthesia was administered around the iliac crest, which was the harvest site. After completion of the procedure and observation of progress, patients returned home on the same day.

All patients received the same standard therapy and post-treatment monitoring. A visual analog scale (VAS) score was used to classify the pain experienced by the patient during BMAC treatment, with a score of 0 indicating no pain and 10 indicating the worst possible pain. Using an 11-point VAS score, pain was classified as none (0), mild (1–3), moderate (4–6), or severe (7–10). After the BMAC injection, patients were allowed to bear their full weight. Moreover, patients were instructed to resume light activities as tolerated and avoid oral painkillers. No other therapeutic interventions were performed, such as bracing and physical therapy. The occurrence of minor or major complications associated with bone marrow harvesting and injection sites was confirmed through a chart review. Minor complications were defined as events that did not require treatment in the operating room. Major complications were defined as events that required treatment in the operating room and subsequently required prolonged unplanned hospitalization. All patients who received BMAC treatment were interviewed during their usual follow-up visits to the orthopedic clinic. After treatment, clinical follow-ups were performed at 2 weeks and 1, 3, and 6 months in both groups and included the assessment of clinical outcomes and re-measurement of pain associated with the procedure, as well as the recording of adverse events. In particular, during the first clinical follow-up, we recorded detailed interviews with patients concerning the pain associated with the procedure. Any patient who did not visit the clinic was contacted via telephone during follow-up evaluations. Two nurses and one private physician identified and visited the non-responders.

### 2.3. Statistical Analysis

The sample size was determined using the G-power program (https://www.g-power.com/en/) [14]. An effect size of 0.5, alpha level of significance of 0.05, and statistical power of 0.8 were considered for sample size analysis. The appropriate sample size for the outpatient group was determined to be 43 people. The outpatient group’s sample size in the present study was 40 people, which may be insufficient. Thus, the propensity score matching method was used to compare the two groups to increase statistical power. Furthermore, statistical tests using the bootstrap method [15] revealed no significant difference between the two groups (Table 1). Through the bootstrap method, we set the confidence interval and estimated the significance level in the re-sampled distribution.

Moreover, propensity score matching was used to compare the basic clinical characteristics of the two groups. Propensity scores were calculated for age, sex, BMI, and K–L grade, and the outpatient and inpatient groups were matched in a 1:2 ratio. Student’s t-test was used to analyze age, BMI, and VAS scores, and the chi-squared test was used to examine sex, K–L grade, and complications. All analyses were conducted using the Statistical Package for the Social Sciences version 18.0 (Chicago, IL, USA). All reported *p*-values were two-sided, and *p*-values < 0.05 were considered to indicate statistical significance.

## 3. Results

The demographic characteristics of outpatient and inpatient groups are summarized in Table 2.

In the outpatient and inpatient groups, pain was moderate or severe and mild or non-existent in 30 (75%) and 80 (100%) patients, respectively, during trocar insertion (mean VAS pain score, 5.2 vs. 1.3; *p* < 0.05) and in 35 (88%) and 80 (100%) patients, respectively, during bone marrow aspiration (mean VAS pain score, 6.2 vs. 1.4; *p* < 0.05) (Table 3). Outpatients experienced more pain during bone marrow aspiration than during trocar insertion (5.2 vs. 6.2, *p* < 0.05). Most patients in both groups experienced mild or no pain upon BMAC injection into the knee (Table 4), with mean VAS pain scores of 2.2 and 2.3 in the outpatient and inpatient groups, respectively (*p* = 0.858).

After BMAC treatment, 7 (17.5%) outpatients and 13 (16.3%) inpatients developed transient post-treatment complications (Table 5). There were no significant differences in the incidence of complications between the two groups (*p* = 0.862), all of which were resolved within 2 months. With conservative treatment, all hematomas resolved within 1 month without sequelae. Numbness (n = 3) recovered within 2 months. One inpatient with obesity had overlapping skin and developed contact dermatitis, which was resolved within 1 month of medication. Seven patients in both groups developed mild-to-moderate swelling at the knee injection site, which resolved within 1 week without any specific measures. Severe swelling and pain were observed on the day of knee injection in one outpatient and two inpatients, who were treated with compression and intravenous analgesics via aspiration. There were no major complications, such as iliac fracture or infection, at the harvest or injection sites in either group.

## 4. Discussion

This study demonstrated that outpatients who received only local anesthesia for BMAC treatment experienced significant pain during trocar insertion and bone marrow aspiration. There were no differences in pain scores during BMAC injection and complications after BMAC treatment between the two groups. Our results imply that bone marrow harvesting for BMAC treatment is painful and requires intravenous anesthesia in addition to local anesthesia for pain control.

Bone marrow aspiration and biopsy (BMAB) is an essential procedure in hematology cases [16], typically performed in an outpatient setting by experienced clinicians. The BMAB procedure is known to be associated with varying degrees of pain and anxiety [17,18]. Several studies have demonstrated that combining intravenous and local anesthesia significantly reduces pain intensity during BMAB. In a randomized controlled trial, Chakupurakal et al. [19] reported superior pain relief and reduced procedural recall after the administration of intravenous midazolam along with local anesthesia. Similarly, Giannoutsos et al. [20] revealed lower pain scores in patients who received local anesthesia and intravenous midazolam than in those who received local anesthesia alone. In this study, many outpatients who received local anesthesia alone reported moderate-to-severe pain during trocar insertion or bone marrow aspiration, whereas all inpatients who received intravenous anesthesia with local anesthesia experienced mild or no pain. These findings highlight that local anesthesia alone may be insufficient for pain control during BMAC treatment, emphasizing the importance of additional intravenous anesthesia.

Thus, a cost-effectiveness analysis of additional intravenous anesthesia should be considered. Given Korea’s national health insurance system, the cost of intravenous anesthesia accounts for a small portion of the overall cost of BMAC treatment; thus, there is minimal difference in the BMAC treatment cost between the outpatient and inpatient groups. Therefore, additional intravenous anesthesia to control pain during BMAC treatment may be cost-effective for patients. However, there may be differences in health insurance systems between countries; thus, future research on determining the cost of BMAC treatment for outpatients and inpatients in major countries is needed.

Complications associated with BMAC treatment can occur at both harvest and injection sites. Bone marrow aspiration, although theoretically safe due to percutaneous procedures, is reported by most hematologists to be associated with complications such as pain, infection, nerve injury, and blood loss. Additional challenges include the limited volume of bone marrow aspirate samples, difficulty in accessibility, and prolonged surgical time [21]. Recently, Baek et al. [11] reported a notable BMAC-related complication rate of 5.3%. Hernigou et al. [22] revealed that among patients undergoing bone marrow aspiration, 1% required narcotic drugs for 24 h due to significant pain, and 0.6% experienced major complications such as deep hematoma requiring blood transfusion or iliac crest fracture. Thus, inserting a trocar needle into the donor’s iliac crest is risky. BMAC is expected to have relatively few complications after injection into the knee because it is fully autologous and does not typically cause foreign body reactions. However, severe knee swelling and pain can occur because of the heparin supplement in BMAC [11]. In this study, one outpatient and two inpatients experienced severe knee swelling and pain on the day of knee injection. Owing to the severity of the pain, inpatients received compression and intravenous analgesia immediately after knee aspiration, whereas an outpatient underwent compression and intravenous analgesia following knee aspiration the next day after experiencing pain upon returning home and was subsequently admitted as an outpatient. Comprehensive strategies for managing complications that occur during BMAC treatment include avoiding nerve damage, applying adequate compression after BM aspiration, avoiding overlapping skin areas at the trocar insertion site, using antibiotics, and applying compression after knee injection. Therefore, the risk of complications during BMAC harvest and knee injection should be considered, and patients should be closely monitored for several days after the procedure.

In this study, outpatients who received local anesthesia alone reported more severe pelvic pain during bone marrow aspiration than during trocar insertion, likely due to the negative pressure generated in the syringe during aspiration. Conversely, inpatients who received intravenous and local anesthesia experienced mild or no pain during trocar insertion and bone marrow aspiration. This indicates that pain is a crucial issue in bone marrow harvesting, and pain management remains a significant concern. Midazolam is commonly used as an intravenous anesthetic agent in our institution and is the preferred benzodiazepine for sedation during medical procedures. However, it has a slow onset of action and prolonged recovery time due to active metabolites. Therefore, achieving adequate anesthesia depth with midazolam can be challenging and may sometimes lead to falls [23]. Additionally, the potential adverse effects of intravenous anesthesia should be considered.

This study has several limitations. First, data were collected prospectively but analyzed retrospectively. Second, the mean patient follow-up periods in the outpatient and inpatient groups were 4.6 and 4.5 months, respectively, both of which are relatively short. Studies with a longer follow-up period are warranted to evaluate the long-term pain, complications, and efficacy of BMAC treatment. Third, the absence of randomization in selecting patients for both groups may introduce selection bias. However, patients were not randomized to accommodate individual circumstances and convenience. Fourth, the single-center design may limit the interpretation and generalization of the findings. Finally, we did not record whether the patients administered oral analgesics or anti-inflammatory drugs, which could hinder the study results regarding pain in both groups. The strength of this study lies in its focus on pain and complications associated with BMAC treatment in outpatient and inpatient settings and under different anesthesia methods.

## 5. Conclusions

To date, there are insufficient evidence-based guidelines for the optimal therapeutic setting and reduction in pain associated with BMAC treatment. This study compared the pain and complications associated with BMAC treatment between outpatient and inpatient groups under different anesthetic methods. This study showed that the pain associated with BMAC treatment was significant among outpatients who received local anesthesia alone, particularly during trocar insertion and even more so during bone marrow aspiration due to the negative pressure exerted by the syringe. To reduce pain associated with BMAC treatment, the addition of intravenous anesthesia to local anesthesia is recommended. Furthermore, it should be fully considered that various complications related to BMAC treatment may occur, necessitating several days of close monitoring.

## Figures and Tables

**Table 1:** Statistical test using the bootstrap method between the two groups.

	95% Confidence Interval	
	Outpatient Group	Inpatient Group
Gender (%)	64–92	74–94
Age	61.75–65.05	62.70–65.35
Body mass index	24.62–26.29	25.23–26.22
K–L grade (%)	0–0	0–0

K–L: Kellgren–Lawrence.

**Table 2:** Patient demographics.

	Outpatient Group	Inpatient Group	*p*-Value
Number of patients (knees)	40 (40)	80 (80)	1.000
Male/female	8:32	16:64	1.000
Age (years) (mean ± SD)	63.4 ± 5.2	64.0 ± 5.9	0.572
Body mass index	25.5 ± 2.6	25.7 ± 2.2	0.552
K–L grade			1.000
II	22	44	
III	18	36	

SD: standard deviation, K–L: Kellgren–Lawrence.

**Table 3:** Pain during the BM harvest procedure on the anterior iliac crest.

	Outpatient Group	Inpatient Group	*p*-Value
1. Pain during trocar insertion			
VAS score (0)	0	22	
VAS score (1–3)	10	58	
VAS score (4–6)	25	0	
VAS score (7–10)	5	0	
Mean VAS pain score	5.2 ± 1.9	1.3 ± 1.1	<0.05
2. Pain during BM aspiration			
VAS score (0)	0	19	
VAS score (1–3)	5	61	
VAS score (4–6)	22	0	
VAS score (7–10)	13	0	
Mean VAS pain score	6.2 ± 2.2	1.4 ± 1.1	<0.05

BM: bone marrow, VAS: visual analog scale.

**Table 4:** Pain during BMAC injection into the knee.

	Outpatient Group	Inpatient Group	*p*-Value
Pain during knee injection			
VAS score (0)	11	21	
VAS score (1–3)	27	57	
VAS score (4–6)	2	2	
VAS score (7–10)	0	0	
Mean VAS pain score	2.2 ± 1.4	2.3 ± 1.5	0.858

BMAC: bone marrow aspirate concentrate, VAS: visual analog scale.

**Table 5:** Post-treatment complications.

	Outpatient Group	Inpatient Group	*p*-Value
1. Anterior iliac crest			
Hematoma	2	4	
Numbness	1	2	
Dermatitis	0	1	
Infection	0	0	
Fracture	0	0	
2. Knee			
Mild and moderate swelling	3	4	
Severe swelling and pain	1	2	
Infection	0	0	
3. Post-treatment complications	7 (17.5%)	13 (16.3%)	0.862

## Data Availability

The raw data supporting the conclusions of this article will be made available by the authors upon request.
